# Maternal Nutrition and Developmental Programming of Male Progeny

**DOI:** 10.3390/ani11082216

**Published:** 2021-07-27

**Authors:** Sarah McCoski, Amanda Bradbery, Rodrigo da Silva Marques, Christian Posbergh, Carla Sanford

**Affiliations:** Department of Animal and Range Sciences, Montana State University, Bozeman, MT 59718, USA; amanda.bradbery@montana.edu (A.B.); rodrigo.marques@montana.edu (R.d.S.M.); christian.posbergh@montana.edu (C.P.); carla.sanford@montana.edu (C.S.)

**Keywords:** bull, developmental programming, growth, ram, reproduction

## Abstract

**Simple Summary:**

The objective of the following review is to describe available literature on the interaction between maternal nutrition and developmental programming in male offspring. The majority of current research focuses on female offspring or fails to take offspring sex into account, though sexual dimorphisms in response to maternal diet are well-recognized. This leaves a large gap in the understanding of male developmental programming. This review will specifically discuss the impacts of maternal dietary energy and protein on bull and ram growth, development, and reproductive capacity in later life.

**Abstract:**

Poor maternal nutrition can cause several maladaptive phenotypes in exposed offspring. While non-sex-specific and female-specific adaptations are well-documented, male-specific outcomes are still poorly understood. Of particular interest are the outcomes in bulls and rams, as developmental programming directly impacts long-term productivity of the animal as well as human food security. The following review discusses the impact of poor maternal dietary energy and protein on bull and ram developmental programming as it relates to growth, development, and reproductive capacity. The review also highlights the importance of the timing of maternal dietary insult, as early-, mid-, and late-gestational insults can all have varying effects on offspring.

## 1. Introduction

Developmental programming is the response to a specific maternal challenge, particularly nutritional, during a critical developmental window that persistently alters the trajectory of growth, physiology, and/or metabolism of the progeny [1,2]. Maternal nutrition during embryonic and fetal development can have short- and long-term consequences for the progeny [3,4,5]. The following review will discuss available literature examining the impacts of maternal energy and protein availability during gestation on subsequent bull and ram growth, development, and reproductive capacity. The review will also highlight the impact timing of the dietary insult has on fetal outcomes.

Worldwide, beef cattle and sheep production systems rely largely on forage-based diets as the source of the majority of nutrients. However, seasonal variation in forage quality and quantity frequently affect nutrient utilization and animal performance by inadequate dietary intake, including energy and protein [6]. Regardless of operation location and environment, cattle herds and sheep flocks have nutritional challenges which require supplementation. However, management practices can vary, especially considering intensive versus extensive operations and differences in parturition timing to meet market needs. Furthermore, changes in nutritional resource availablity, as in drought years, or projected market values for a calf or lamb crop may cause producers to inadequately supplement their gestating animals due to financial restraints or inability to source feedstuffs. Thus, beef cattle and sheep are often exposed to suboptimal nutrition during critical periods of fetal development, which consequently alter metabolic, energetic, and body composition response of the offspring [2,5,7]. To maintain maximum long-term productivity, producers must design and integrate supplementation programs, such as those focused on protein and energy, according to animal requirements and forage characteristics. The effects of dietary manipulation during gestation on offspring outcomes might vary depending on the timing, severity, and duration of nutrient restriction or overnutrition, as well as the sex of the fetus [8,9,10]. A limited number of studies have investigated the combined influence of maternal diet and fetal sex on offspring performance, metabolism, and health. Those that have included fetal sex in analyses, a majority are focused on female offspring because of their importance as replacement animals. The work discussed herein is focused on the interaction between maternal energy and protein availability during gestation on male offspring growth, development, and reproductive parameters in cattle and sheep. The purpose of this review is to highlight available information on this topic, but also to bring attention to the lack of understanding on how male offspring are impacted by maternal diet. 

The loss of quality and quantity of available forage during gestation often results in a state of nutrient restriction and may alter the intrauterine environment of the developing offspring. While undernutrition is more common in livestock production systems, overnutrition presents a similar concern, though the effects are not as well defined. Regardless, when exposed to changes in maternal plane of nutrition *in utero*, offspring are at greater risk of developing metabolic dysregulation. While little work has been performed with the intent to compare the effects of maternal plane of nutrition and fetal sex, it is an important consideration given the evidence of sexual dimorphism in fetal development [11,12]. This may be especially true with regards to skeletal muscle and adipose tissue development, as well as postpubertal reproductive efficiency. Hence, the focus of this review is to summarize recent advancements in the understanding of interactions between maternal dietary management and growth, performance, and reproductive capacity of male offspring.

## 2. Maternal Under- and Over-nutrition Impacts on Skeletal Muscle and Adipose Tissue Development

**Figure 1:**
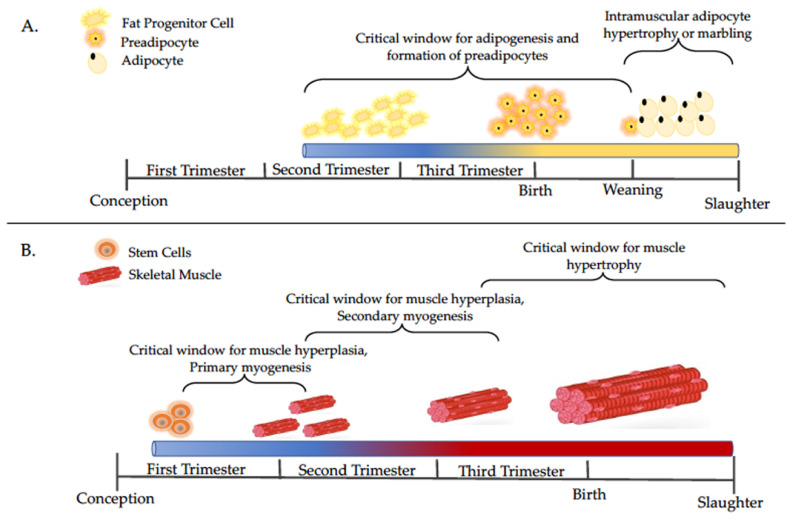
Timelines of adipocyte (**A**) and muscle (**B**) development with a focus on major events discussed in this review. Adapted from [14,17,18].

Maternal nutrient status during gestation is critical for fetal skeletal muscle and intramuscular adipocyte development, which can be a determinant factor of the future performance and carcass characteristics of the progeny [13,14,15]. The impacts of maternal diet on offspring growth and performance are variable and dependent on the timing of the dietary intervention, as muscle and adipose development occur at specific times in gestation (see Figure 1A,B). During the prenatal stage, the formation of muscle fibers can be altered by maternal nutrient restriction, leading to a reduction in the total number of secondary muscle fibers, which permanently reduces animal performance [13]. Conversely, maternal overnutrition promotes the preeminent expression of adipogenic genes in fetal skeletal muscle [16], which likely program a greater number and size of adipocytes of progeny skeletal muscle postnatally [15]. Therefore, maternal nutritional status during fetal development is a major factor affecting the lifetime productivity of the offspring [2,4,5].

Undernutrition often results in offspring that are small for gestational age, culminating in smaller birth weights followed by a compensatory response during postpartum growth in sheep and cattle [19,20,21]. The observed reduction in birth weights may be due to a prioritization of vital organs over the development of muscle mass in animals that are exposed to nutrient restriction *in utero*. However, weight and mass of some fundamental organs involved in metabolism and growth are also known to be sensitive to maternal plane of nutrition including the pancreas, liver, intestines, skeletal muscle, adrenals, etc. [22,23,24,25]. Much of the resulting postpartum compensatory growth is due to altered offspring metabolism from multifactorial changes at the molecular level of several organ systems [19,20,24,25]. 

Despite the “catch-up” growth often observed following fetal exposure to maternal nutrient restriction, offspring tend to have higher levels of adiposity and less lean mass compared to offspring whose dams were fed to meet nutrient requirements during gestation [14,26,27,28]. Adipose and skeletal muscle tissues are both derived from the mesenchymal stem cell populations (Figure 1) thus, the effects of maternal nutrition on the differentiation of one tissue will also influence the differentiation and development of the other [15]. The mechanism by which this occurs is still under investigation; however, an interplay of molecular alterations appears to be implicated. Results describing molecular-specific effects vary based on timing of dietary manipulation, but the long-term implications are similar.

Increased adiposity and decreased lean mass in response to maternal overnutrition are a product of changes in gene expression in both adipose tissue and skeletal muscle. Because skeletal muscle is the primary site of insulin-stimulated glucose uptake, it is a prime target for altered metabolism resulting in disturbances that contribute to the deviations in offspring growth and development. Reduced insulin-like growth factor 1 and 2 (IGF1/2), due to maternal nutrient restriction, with upregulation of their respective receptors on skeletal and cardiac muscle has been reported, which is associated with insulin dysregulation [24,29]. Other studies do not report the same effects, but instead observed differences in gene expression and signaling molecules in other metabolic pathways including adipogenic, lipogenic, and myogenic factors [30,31,32]. Additionally, Sandoval et al. [24] found alterations in metabolic pathways in skeletal muscle and a lower proportion of type I skeletal muscle fibers, which are the most sensitive of the myofiber types to insulin and oxidative metabolism, further contributing to altered energy metabolism, growth, and development [24,25]. None of these data, however, infer a difference between fetal sex in response to maternal plane of nutrition in developing lambs.

Differences between fetal sex response to maternal undernutrition is variable, and a critical gap remains in the scientific literature. It is suggested that male offspring may be more susceptible to maternal nutrient restriction because their energy demands for development may be higher [12,33]. Ford et al. [26] found glucose intolerance in male lambs following a glucose tolerance test at 12 months of age following exposure to maternal undernutrition; however, the study did not compare male and female lambs. Similarly, beef steers exposed to different levels of nutrient restriction *in utero* had increased average daily gain consistent with compensatory growth, but heifer offspring were not included in the study [21].

Although undernutrition often results in reduced birth weights, the same is not always the case with maternal overnutrition where birth weights tend to be similar between offspring exposed to maternal overnutrition or control diets meeting nutrient requirements [22,23,34]. The influence of maternal overnutrition on organ weights and mass is not as predictable as that observed with nutrient restriction, although many similar molecular changes to organ systems have been described [23,34,35,36,37]. The influence of maternal overnutrition specific to the development of male offspring is not well investigated; however, sex-specific responses are becoming more frequently considered. 

Tissue-specific responses to maternal overnutrition include the pancreas, skeletal muscle, adipose tissues, and intestines, resulting in reduced insulin sensitivity, increased adipogenesis, and altered growth and performance [23,26,28]. Several genes and biochemical pathways have been implicated in these responses; however, they may differ based on fetal sex and timing and duration of the nutritional insult. A comparison of male and female offspring revealed that male offspring prioritize skeletal muscle development over intestinal development compared to female offspring when exposed to maternal overnutrition *in utero* [34,35]. Furthermore, male offspring tend to develop skeletal muscle faster than female offspring, which could alter the response to maternal overnutrition and the resulting performance phenotypes [34,35]. Future work should focus on elucidating sex × tissue-specific responses to maternal nutritional status during gestation.

## 3. Maternal Dietary Energy

### 3.1. Maternal Dietary Energy and Developmental Programming

Most energy models available consider the energy required for the first months of gestation to be negligible due to minimal energy retention in the pregnant uterus, whereas with the progression of the gestation energy requirements and retention in the gravid uterus is maximized to support fetal growth and development [6,38]. Net energy for maintenance increases by 30–50% during the last trimester of gestation, of which half of the increase contributes to gravid uterine tissues and one-quarter to the fetus to support rapid fetal growth and one-quarter for increased maternal metabolic function [6,38]. Nevertheless, the early stage of embryo differentiation, fetal organogenesis, and utero-placental tissues formation are critical events that impact progeny later in life [4,5,38,39]. Accordingly, ewes subjected to a 50% nutrient restriction during early gestation and then realigned to meet energy requirements produced lambs with normal birth weights, whereas lambs from these restriction-realigned dietary regimen ewes exhibited altered growth rates, dysfunction in glucose metabolism, increased carcass fat, and reduced carcass muscle compared with lambs from ewes offered a control diet throughout gestation [26,27,39]. Therefore, dams that undergo nutritional stress during early period of development, rather than late gestation, are expected to calve a normal weight offspring that will still experience undesired growth and metabolic problems later in life [26,27,38,39]. These findings contradict the common misconception that maternal nutrition during early gestation is less critical than that during mid- and late gestation. Additionally, it should be noted that these studies did not take offspring sex into account. Similar studies should be performed to determine if these results vary depending on offspring sex. 

Maternal nutrient status affects nutrient partitioning of pregnant livestock and fetal development [38,40]. This may disturb the glucose and amino acid metabolism of the gravid uterus, and these nutrients are the primary source of energy for the normal development of the fetus [41,42]. In sheep, Gardner et al. [43] demonstrated that a 50% restriction in energy during late gestation did not impact lamb birth weight, but decreased glucose tolerance, caused insulin resistance, and increased adiposity in lamb progeny. Long et al. [28] reported that 30% of energy restriction during early gestation in beef cows did not impact birth, weaning, or carcass characteristics, but adipocyte diameter was increased, and muscle weight was reduced in the male progeny born to restricted cows. Corah et al. [44] demonstrated that calves from beef cows offered an energy-restricted diet during the last 100 days of gestation were lighter at birth and weaning compared with calves from cows fed 100% of energy requirements. Furthermore, energy deficiency during the last trimester of gestation increased rates of morbidity and mortality of the progeny [44], suggesting that maternal energy status during gestation can also impact the health of the offspring. In an effort to explain the mechanism by which these phenotypic changes occur, Sanglard et al. [43] evaluated the skeletal muscle and blood transcriptomes of beef calves that were exposed to energy restriction in utero. Authors describe effects of diet, sex, and diet × sex interactions on expression of genes involved in energy metabolism, skeletal muscle development and contraction, immune response, and responses to stress. These effects emphasize the sex-specific response of offspring to maternal energy status [43]. 

Source of maternal dietary energy may also promote adaptations that permanently alter the trajectory of growth, physiology, and metabolism of the progeny. Loerch et al. [45] indicated that calves from cows limit-fed a starch-based diet have greater birth weights compared with calves from cows fed a forage-based diet. Radunz et al. [46] offered hay-, corn-, or distillers grains-based diets to late-gestating beef cows and observed a reduced birth weight in calves from hay-fed cows compared with cohorts born to cows receiving corn or distillers grains, whereas calves from hay-fed cows were lighter at weaning and required 10 more days on feed to reach a similar fat thickness compared with calves from corn-fed cows. These outcomes suggest that high-starch diets offered to gestating cows alter rumen fermentation towards propionate production, which may increase the circulating blood glucose and insulin secretion [47] resulting in greater maternal nutrient supply to the fetus growth [48,49]. Collectively, nutritional energy manipulation during periods of developmental plasticity such as the embryonic, fetal, and neonatal periods exert lasting effects on muscle and adipose tissue development, health, and overall performance of offspring. It should be noted that these specific studies did not take offspring sex into account. Further studies are necessary to determine these impacts on bull and ram offspring.

### 3.2. Dietary Energy In Utero and Male Reproductive Capacity

**Figure 2:**
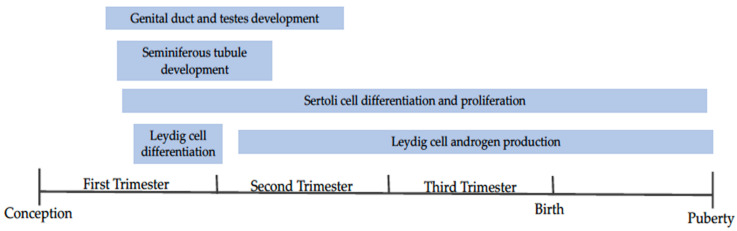
A timeline depicting the development of the bull and ram reproductive tracts. Events included are discussed within the review. Adapted from [58,59,60].

Testes size has long been used as an indicator of fertility, as scrotal circumference is directly related to semen production capacity [50,51,52]. Scrotal size can change based on nutritional status, breeding season, and age of the animal thus, it seems logical that maternal nutrition during testes development (beginning on day 41 and 31 post-fertilization in bull and ram embryos, respectively [53,54]) can also impact testes size and weight (Figure 2). Studies show varying results, likely an effect of the differing times of dietary intervention. Ram lambs from ewes fed to 70% of energy requirements from week 10 of gestation to birth had lower paired testes weight compared to those exposed to 110% energy requirements [55]. Similar results were reported when ewes were restricted to 50% energy requirements from day 100 until parturition, with ram lambs experiencing reduced testicular weight at birth [56]. Conversely, ram lambs from ewes fed 50% energy requirements from breeding to day 95 of gestation showed no effect on testicular weight compared to controls [57]. These differences in results may be explained by the timing of the nutritional insult. Nutrient restriction encompassing the entirety of testes development may result in more profound impacts on testes formation than restrictions ending during early/mid development. Scenarios such as the later, may give the testes enough time during development to “catch up” to normal growth. Additionally, the timing of nutritional insult may differentially impact the development of various testicular structures (i.e. seminiferous tubules, Sertoli cells, vasculature, etc.) which may result in differing phenotypic changes to testes structure. 

Sertoli cell number and function may be used to assess male reproductive capacity, as a single Sertoli cell is able to support the development of a finite number of sperm cells within the seminiferous tubule [61], play a role in male sex determination [62], and produce secretions necessary for spermatogenesis. Early in embryogenesis, Sertoli cells secrete Mullerian inhibiting factor, which prevents the differentiation of the female reproductive tract [63]. Additionally, the number of Sertoli cells dictates the total number of germ cells in adulthood, as Sertoli cells secretions are indispensable in promoting spermatogenesis [61,64,65]. Studies highlight the impacts of poor maternal nutrition on offspring Sertoli cell number. A study utilizing sheep to examine the impacts of reduced metabolizable energy and crude protein (50% vs 100% requirement) found that males exposed to energy restriction from days 31–100 of gestation had lower Sertoli cell numbers than controls, though testes weight did not differ between groups [66]. Additional work reported similar findings in which maternal energy restriction reduced Sertoli cell and seminiferous tubule numbers in ram lambs [55,56]. However, these studies are contradicted by work which found ram fetuses exposed to low energy (50% requirement) maternal diets had unaltered Sertoli cell number [67]. Collectively, these findings indicate that the timing of the maternal dietary insult and when the effects of maternal dietary insults are assessed are important to consider. Sertoli cell numbers increase drastically from mid-gestation to birth in sheep and cattle (reviewed in [59]). Considering this, implementing a maternal dietary insult during this window may produce different results compared to those following maternal dietary intervention during early gestation. Additionally, Sertoli cell numbers continue to increase from birth through the peripubertal period. Sampling prior to this window of Sertoli cell proliferation [55,67] may yield different results than studies in which samples are collected during or after this period. The effect of timing should be investigated further. The impacts of maternal undernutrition on Sertoli cell development is not fully understood. Sertoli cells are an undisputed necessity in male reproduction, and disruption in their development and function has damaging effects on male fertility. However, it is important to recognize that Sertoli cells continue to proliferate after birth, therefore, a sufficient postnatal diet may recover the damaging effects of gestational dietary insults. The biological impacts of altered Sertoli cells during early life on reproductive performance in adulthood remains unclear. 

The mechanisms resulting in the structural differences in the gonads of males exposed to maternal high or low dietary energy are still not fully understood. There are two possible causes for the connection between maternal diet and later reproductive capacity in male offspring. First, the effects discussed above may be the direct consequence of nutrition on the gonads, as made evident by gene expression data. Bull fetuses exposed to maternal overnutrition experienced a decrease in expression of steroidogenic acute regulatory protein (StAR), hydroxysteroid 17-Beta dehydrogenase 3 (HSD17B3), IGF1, IGF2, and IGF1 receptor (IGF1R), genes involved in testes development and steroidogenesis [68]. In particular, IGF1 is produced by Leydig and Sertoli cells, and IGF1 receptor (IGF1R) is found on germ and somatic cells of the testis [69]. When inactivated in mouse testes, decreased IGF1R resulted in a decrease in Sertoli cell proliferation [70]. As previously discussed, Sertoli cells play an indispensable role in testes development and later reproductive capacity, and alterations in their proliferation may have lasting impacts on male reproductive performance. A second possibility is that the observed gonadal effects are secondary to disruptions in hypotholamo-pituitary function. Studies show that exposure to maternal energy restriction alters pituitary sensitivity to gonadotropin releasing hormone (GnRH), which may disrupt gonadotropin release [71,72]. Seminiferous tubule function and Sertoli cell development are dependent on gonadotropin production during fetal development [73] thus, a reduction in GnRH responsiveness may result in reproductive insufficiency in adult life. Further work is needed to identify the mechanisms responsible for the link between maternal dietary energy availability and male offspring reproduction in later life.

Limited information is available on the impacts of in utero growth-restriction on adult reproductive parameters in bull and ram offspring. A study utilizing ewes fed either a normal or a growth restricted diet found that male offspring experienced delayed puberty and had lower plasma testosterone and testicular volume until 28–35 weeks of age [74]. Furthermore, though peak testosterone production occurred at a similar age, the growth-restricted males saw a much lower peak value, 5.8 ± 1.1 versus 9.4 ± 0.5. Puberty was not attained in these rams until live weights similar to those of the control rams at puberty was achieved. The delay in the onset of puberty in the growth-restricted male lambs indicates a significant impact in maternal diet and the onset of sexual maturation in sheep [74]. Rams and bulls that attain puberty at an earlier age compared to their counterparts allow for earlier progeny testing, increase the rate of genetic selection, and can reduce production costs for breeders. These findings suggest that male reproductive capacity is susceptible to developmental programming; however, studies examining testicular structure and semen quality during adult life are necessary.

## 4. Maternal Dietary Protein

### 4.1. Dietary Protein and Developmental Programming 

As previously mentioned, supplementation is often required in livestock production systems based typically on forage diets, and protein is often considered the limiting nutrient in livestock operations [75]. Research has suggested protein supply during gestation affects fetal, growth, and development, and is critical to offspring performance and health [76,77,78,79]. The crucial role of specific amino acids on cell metabolism and function of the fetus might exert lasting effects on muscle and adiposity development, health, and overall performance of the progeny [77,80,81]. For example, arginine is a conditionally-essential amino acid that contributes to nitric oxide and polyamine production, both of which play important roles in placental development and function [82,83,84]. Therefore, it seems reasonable that maternal dietary protein supply during gestation is indispensable for adequate progeny trajectory prenatally and postnatally. 

A growing body of evidence suggests that maternal protein nutrition might alter offspring body composition and growth, hormonal balance, metabolic function, neonatal health, organ development, and function [2,85,86]. Maternal undernutrition during early gestation, which is a critical window for establishing normal fetal development of all organs and tissues [87,88], leads to reduced fetal muscle mass and increased adipocyte size that could modify progeny metabolism later in life [13,85]. Accordingly, Copping et al. reported that pregnant heifers exposed to low dietary protein during the first trimester of gestation affected the development of the fetal heart, liver, lung, brain, and pancreas with sex-specific responses to the gestational dietary protein restriction [10]. Specifically, maternal protein restriction during first trimester gestation resulted in differential gene expression in the liver between male and female offspring [62]. Male offspring had increased expression of glucose transporter protein type 1 (GLUT1) over female offspring, whereas female offspring more greatly expressed peroxisome proliferator-activated receptor γ (PPARγ) with reduced expression of IGF2 and glucocorticoid receptor. This suggests altered metabolism between the sexes, which would both result in dyslipidemia and increased adipogenesis postnatally, but programmed through different molecular mechanisms based on fetal sex [62]. Sex-specific cardiovascular and central nervous system effects have also been observed in cattle and sheep which are believed to further drive metabolic differences through the preferential allocation of nutrients during periods of protein restriction [10,89,90]. 

Maternal nutrient intake affects fetal growth trajectory from early stages of fetal development to birth. A large proportion of available data is focused on late gestation when nearly 75% of the fetal growth occurs, and nutrient requirements for fetal development are maximal [6,28]. Furthermore, variations in maternal dietary protein might alter the performance and carcass characteristics of male progeny reared for slaughter [7,78,91,92]. Funston et al. [93] reported that protein supplementation during gestation has later life effects on the male offspring, including weaning weight and carcass characteristics compared with non-supplemented cohorts. Bohnert et al. [76] also observed that calves from cows supplemented with dried distillers grains with solubles during the last trimester of gestation were heavier at weaning compared with un-supplemented cohorts. Stalker et al. [94] and Larson et al. [78] reported increased weaning and carcass weights in steers from protein-supplemented dams compared with un-supplemented cohorts. Supplying protein to late-gestating cows has also been shown to enhance the proportion of carcasses graded USDA choice and marbling score of the male progeny [78]. Taken together, these findings indicate that dietary protein during gestation impacts fetal growth and development [2], leading to short- and long-term consequences on performance and carcass characteristics of male progeny [76,78,94]. Furthermore, maternal protein status during the gestation affects metabolic and endocrine function of the fetus, which might be programming the offspring to exhibit undesirable productivity, as well as diseases, later in life [2,14,84,85]. Nonetheless, maternal nutrient status for other macronutrients may exert additional impacts on male progeny performance trajectory, though this requires further investigation.

### 4.2. Dietary Protein In Utero and Male Reproductive Capacity

Seminiferous tubules are the site of spermatogenesis within the testicles, and they contain both spermatogenic cells and somatic Sertoli cells. As they are the sole site of spermatogenesis in the male, changes in their length or diameter may alter spermatozoa output by altering the area available for spermatogenesis to occur. Interestingly, seminiferous tubule development is shown to be sensitive to nutritional insults *in utero*. At 98 days of gestation, bull fetuses of dams exposed to a low protein peri-conception diet showed an increase in the proportion of seminiferous tubules and a decrease in blood vessel area, but did not exhibit an effect on the tubule area in the testis. [95]. This indicates protein insufficiency may impact testicular development in a cell-specific manner. 

The plasticity of seminiferous tubule development is highlighted in scenarios of protein over-supplementation. Bull calves exposed to over-supplementation during gestation had a tendency to have a smaller tubule diameter than those exposed to reduced protein levels [96]. These findings compliment a later report of reduced seminiferous tubule length and diameter, and a reduced percentage of testicular tissue made up of seminiferous tubules in calves exposed to overnutrition, though no difference in testicular weight was detected [68]. It is possible that this reduction in seminiferous tubule diameter affects sperm output in these males, however, postnatal assessments are needed to confirm this idea.

Few reports exist on the impacts of low protein exposure in utero on reproductive parameters in mature rams and bulls. One study examining the impact of a low protein diet fed to nulliparous crossbred (Bos taurus x Bos indicus) heifers for 60 days prior to insemination, found male offspring had an increased proportion of seminiferous tubules, decreased blood vessel area in the testis [95]. Additionally, bulls from dams fed a low protein peri-conception diet experienced delayed attainment of puberty compared to their counterparts and had lower sperm quality at day 598 of age [95]. Seasonal variations and occurrence of drought-stricken range results in decreased forage quantity and in low-quality forages. Therefore, protein restriction during peri-conception and first trimester may be more prevalent in both sheep and cattle than once thought. Further research is needed to determine the mechanisms by which protein restriction impacts male offspring reproductive parameters. Future research may assist producers identify the most critical timepoints of developmental programming and allow for more strategic supplementation to ewes and cows to maximize their offspring’s potential.

## 5. Conclusions

Despite many advancements in developmental programming, sex-specific impacts on male offspring growth and reproductive performance remain largely undetermined. This is partially due to the variable results between studies by different types and timing of dietary insults during critical periods of embryonic and fetal development. Maternal undernutrition during early gestation may result in offspring with differing phenotypes than those exposed to the same nutritional insult during mid-late gestation. Tissue and organ system development is coordinated by a highly regulated interplay of multiple biological mechanisms. Accordingly, the effects of inadequate maternal nutrition span a multitude of systems influencing offspring health and production in postnatal life. Both maternal under- and overnutrition have resulted in a shift in favor of adipose development over skeletal muscle, impacting carcass characteristics and nutrient metabolism. Additionally, the same nutritional insults *in utero* have resulted in altered testicular size, Sertoli cell numbers, and seminiferous tubule morphology which may have long-term implications on reproductive efficiency. These findings expose the effects of a single nutritional insult on multiple organ systems which persist into postnatal life. Understanding the effects of maternal nutrition on male offspring development will benefit the development of management practices to optimize growth and postpubertal reproductive efficiency. Furthermore, new knowledge on paternal effects of developmental programming is being revealed, supporting the need to focus on male offspring development like the emphasis already placed on female development. Providing optimal maternal nutrition during all stages of embryonic and fetal development is imperative for normal growth, health, and reproductive performance in postnatal life.
